# Comparison of Adhesion Performance of a Self-curing and a Light-curing Universal Adhesive to Various Dental Substrates and CAD/CAM Materials

**DOI:** 10.3290/j.jad.b4908469

**Published:** 2024-01-26

**Authors:** Cristina M. P. Vidal, Erica C. Teixeira, Steven R. Armstrong, Fang Qian

**Affiliations:** a Assistant Professor, Department of Operative Dentistry, College of Dentistry and Dental Clinics, The University of Iowa, Iowa City, IA, USA. Idea, hypothesis, experimental design, performed the experiments, wrote and proofread the manuscript.; b Associate Professor and Head of Operative Dentistry, Department of Operative Dentistry, College of Dentistry and Dental Clinics, The University of Iowa, Iowa City, IA, USA. Idea, hypothesis, experimental design, performed the experiments, wrote and proofread the manuscript.; c Professor Emeritus, Department of Operative Dentistry, College of Dentistry and Dental Clinics, The University of Iowa, Iowa City, IA, USA. Idea, hypothesis, experimental design, proofread the manuscript.; d Associate Research Scientist, Division of Biostatistics and Bioinformatics, Iowa Institute for Oral Health Research, College of Dentistry and Dental Clinics, The University of Iowa, Iowa City, IA, USA. Performed statistical evaluation, proofread the manuscript.

**Keywords:** universal adhesives, bond strength, resin cement, enamel, dentin, self-curing adhesive, CAD/CAM

## Abstract

**Purpose:**

To compare the adhesion of a self-curing (Tokuyama Universal Bond, TUB) and a light-curing (Scotchbond Universal, SBU) universal adhesive to CAD/CAM materials, enamel, and dentin. This study also assessed differences in enamel adhesion between self-etch vs selective etching modes, as well as immediate and long-term adhesion to dentin for both adhesives.

**Materials and Methods:**

Shear bond strength (SBS) testing was used to assess adhesion to enamel, dentin, Lava Ultimate (LU), Vita Enamic (VE), IPS e.max CAD (LD), IPS e.max ZirCAD (3Y-Zir), and Lava Esthetic (5Y-Zir) (n = 10). Moreover, bonding to enamel in self-etch and selective etching modes (n = 10) as well as immediate and aged resin-dentin bond strength (24 h after bonding, after 100,000 thermal cycles [TC] and long-term storage) was evaluated using the microtensile bond-strength test (n = 30). Failure mode was also determined for the bonding to dentin. Statistical analyses consisted of one-way and two-way ANOVA with appropriate post-hoc Tukey-Kramer or two-sample t-tests, as well as the chi-squared or Fisher’s exact test (α = 0.05).

**Results:**

TUB and SBU universal adhesives presented similar bonding to LU, VE, 3Y-Zir, and 5Y-Zir. However, SBS for TUB was superior to SBU when bonding to lithium-disilicate glass-ceramic (IPS e.max CAD). SBU showed better adhesion to dentin and enamel when used in the self-etch mode, while TUB promoted strong bond strength to enamel in the selective etching mode. TUB after TC was the only aging condition that yielded a significant reduction in resin-dentin bond strength.

**Conclusion:**

In-vitro adhesion performance of the self-curing and light-curing universal adhesives varies depending on the dental substrate or CAD/CAM restorative material used for bonding.

Universal adhesive materials have continuously evolved and become popular due to their ease to use with different bonding strategies. Recent evidence from clinical trials reports satisfactory performance of universal adhesives when used for bonding to non-carious cervical lesions.^11,12,28^ Universal adhesives are less technique sensitive, more user-friendly, involve fewer application steps, are versatile, and promote adhesion to several different substrates and materials depending on their formulation.^2,10,12,21^ Due to the presence of phosphate acidic monomers such as 10-methacryloyloxydecyl dihydrogen phosphate (10-MDP) and silane, universal adhesives can be used for bonding to zirconia and various silica-based ceramics in a simplified protocol that should not require the application of additional primers.^9,20,21,31^

Aiming for further simplification of the clinical protocol, self-curingd bonding systems, such as Tokuyama Universal Bond (TUB), which does not require light activation, were introduced on the market a few years ago. This two-bottle universal system presents a novel polymerization technology based on the reaction of an aryl borate catalyst with the functional monomer to form a borane compound. The peroxidase present in the formulation oxidizes the borane, resulting in radicals that serve as initiators of chemical polymerization.^25^ According to the manufacturer, the borate initiator in TUB is a very effective initiator of chemical polymerization due to its high catalytic activity under very acidic conditions. An experimental version of an adhesive containing a borate initiator showed a higher degree of conversion than a camphorquinone-based material in a previous study.^30^ Therefore, besides the ease of application, a self-curing universal adhesive could offer other clinical advantages, including eliminating or reducing the problem of a lower degree of conversion of the bonding materials in areas when irradiation is attenuated and/or restorative materials are thick/opaque.^19,38^ Moreover, incompatibility between the initiator system present in the cement (or restorative material) and residual acidic resin monomers that remain in the oxygen-inhibited layer is a well-described problem with simplified adhesives used in combination with dual-curing resin cements and core build-up resin composites.^36^ This incompatibility leads to substantial reduction of bond strength, inferior mechanical properties of the adhesive and restorative materials, and poor quality of the adhesive and hybrid layers, all of which impair the longevity of the restoration.^39^ While some adhesives present dual-curing or self-curing activators, incompatibility may still be a concern and material dependent.^27,32^ Whereas it is not recommended to combine different adhesives and dual-curing resin-based materials from different companies, incompatibility is often seen even when products from same manufacturer are used together,^27^ possibly due to discrepancies in the pH and acidity of different systems. Lastly, the clinical protocol for bonding an indirect restoration to enamel and dentin may or may not require light curing of the universal adhesive applied on the dentin or on the restorative material,^3,22^ which causes confusion among clinicians. A self-curing adhesive system could potentially eliminate these issues, simplifying the clinical protocol and reducing incompatibility with other dual-curing materials.

In addition, as already mentioned, some universal adhesives already contain silane in the attempt to avoid the additional step of applying a separate silane coupling agent. However, the literature shows that silanes are sensitive to thermal aging and self-condensation, and can be hydrolyzed in the presence of hydrophilic monomers and in low pH universal adhesives,^14,42^ resulting in silane degradation. The manufacturer claims that TUB is provided in two separate bottles to avoid combining the acidic monomer with the ceramic primers and prevent deterioration of the silane coupling agent, which ensures adequate bonding to silica-based ceramics. However, limited in-vitro evidence shows that additional light-curing of a self-curing universal adhesive improved immediate bonding to feldspathic ceramic, but resulted in inferior adhesion to zirconia in comparison to a light-curing adhesive.^25^

Aside from all the advantages of a universal adhesive and the possible lack of incompatibility with dual-polymerizing resin-based materials, it is important to ensure that this new self-curing adhesive presents good performance when bonding to enamel and dentin. As widely reported for self-etching and universal adhesives, selective etching of enamel is mandatory for adequate enamel demineralization, adhesive infiltration, and achieving good interfacial quality.^7,13,33^ However, a very limited number of studies investigated the performance of self-curing universal adhesives and their performance when used with different restorative materials, dental substrates, and bonding strategies.^25^ Therefore, the goals of this in-vitro study were to: 1) evaluate the immediate performance of the TUB adhesive in comparison to a light-curing universal adhesive (Scotchbond Universal, SBU) when used for bonding to different substrates and restorative materials; 2) determine the performance of the TUB and SBU adhesives when applied on enamel using the selective etching or self-etching mode; and 3) compare the stability of TUB and SBU adhesives in resin-dentin interfaces subjected to simulated aging. The null hypotheses tested were that: 1) there is no difference between SBU and TUB in terms of bond strength of resin cements to different restorative materials and dental substrates; 2) there is no difference in the bond strength among the different restorative materials and dental substrates for each of the universal adhesives; 3) there is no difference in the bond strength of resin composite to etched (selective etching) and non-etched (self-etch mode) enamel when bonded with SBU or TUB; and 4) there is no difference in the adhesion performance of SBU and TUB to dentin when comparing immediate and simulated aging conditions.

## Materials and Methods

### Restorative and Adhesive Materials

The following adhesives and resin cements were used in this study: the adhesives Tokuyama Universal Bond (TUB) (Tokuyama; Tokyo, Japan) and Scotchbond Universal (SBU) (3M Oral Care; St Paul, MN, USA) (Table 1), the resin cements Estecem II (Tokuyama) and RelyX Ultimate (3M Oral Care) (Table 2), and the resin composites Omnichroma (Universal Shade, Tokuyama) and Filtek Supreme Ultra (Shade A2E, 3M Oral Care) (Table 2). Moreover, five CAD/CAM materials were used, including a composite resin nanoceramic (Lava Ultimate, 3M Oral Care) (LU), polymer infiltrated ceramic network (Vita Enamic, Vita Zahnfabrik; Bad Säckingen, Germany) (VE), lithium-disilicate glass- ceramic (IPS e.max CAD, Ivoclar Vivadent; Schaan, Liechtenstein) (LD), 3 mol.% yttria partially stabilized tetragonal zirconia (IPS e.max ZirCAD, Ivoclar Vivadent) (3Y-Zir), and 5 mol.% yttria cubic zirconia (Lava Esthetic Fluorescent Full-Contour Zirconia, 3M Oral Care) (5Y-Zir) (Table 2).

**Table 1:** Composition of adhesive systems (with application technique) used in this study

Material, manufacturer	Composition	Application technique
Tokuyama Universal Bond (TUB) Tokuyama; Tokyo, Japan	Liquid A: new 3D-SR monomer (phosphoric acid monomer), bis-GMA, TEG-DMA, HEMA, MTU-6, acetone Liquid B: acetone, isopropyl alcohol, water, acryl borate catalyst, γ-MPTES, peroxide pH 2.2	Dry dentin surface by blotting. Dispense one drop each of TUB A and B into the disposable mixing well and mix thoroughly with a disposable applicator for 10 s. Apply TUB to the entire adherent surface(s) for a total of 5 s. Apply a weak air stream continuously to the TBU surface until the runny adhesive stays in the same position. Finish with a mild air stream to the surface.
Scotchbond Universal Adhesive (SBU) 3M Oral Care; St Paul, MN, USA	MDP phosphate monomer, HEMA, bis-GMA, Vitrebond copolymer, silica fillers, ethanol, water, camphorquinone, dimethylaminobenzoate(-4), silane, dimethacrylate resins pH 2.7	Dry dentin surface by blotting. Apply the adhesive to the prepared tooth and rub it in for 20 s. Gently air dry the adhesive for approximately 5 s to evaporate the solvent. Light cure for 10 s.

Bisphenol A diglycidyl ether dimethacrylate (bis-GMA); 2-hydroxyethyl methacrylate (HEMA); 10-methacryloyloxydecyl dihydrogen phosphate (MDP); 6-methacryloyloxyhexyl 2-thiouracil 5-carboxylate (MTU-6); triethylene glycol dimethacrylate (TEG-DMA); γ-mercaptopropyltriethoxysilane (γ-MPTES).

**Table 2:** Restorative materials (including resin composite and CAD/CAM materials) and resin cements used in the adhesion studies

Material	Classification	Composition	Manufacturer
Omnichroma	Resin composite	UDMA, TEG-DMA, spherical silica-zirconia fillers	Tokuyama (Tokyo, Japan)
Estecem II	Resin cement	Part A: bis-GMA, TEG-DMA, bis-MPEPP, silica-zirconia filler Part B: bis-GMA, TEG-DMA, bis-MPEPP, silica-zirconia fillers, camphorquinone, peroxide	
Filtek Supreme Ultra Universal Restorative	Resin composite	Bis-GMA, UDMA, TEG-DMA, bis-EMA-6, PEG-DMA, phenyl bis(2,4,6-trimethylbenzoyl)-phosphine oxide, silane-treated ceramic, silane-treated silica, silane-treated zirconia	3M Oral Care (St Paul; MN, USA)
RelyX Ultimate	Resin cement	Base paste: methacrylate monomers, radiopaque silanated fillers, initiator components, stabilizers, rheological additives Catalyst paste: methacrylate monomers, radiopaque alkaline fillers, initiator components, stabilizers, pigments, rheological additives, fluorescent dye, dark cure activator for SBU	
Lava Ultimate	Resin nanoceramic	Nanoceramic particles (zirconia filler) (80% by weight), silica filler, aggregated zirconia/silica cluster filler, highly cross linked (methacrylate-based) polymer matrix, silane	
Lava Esthetic	Zirconia ceramic	5 mol% yttria cubic zirconia, esthetic fluorescent full-contour zirconia	
Vita Enamic	Polymer-infitlrated ceramic network	Feldspathic-based ceramic network (86% by weight) infiltrated by acrylate polymer network (UDMA, TEG-DMA) (14% by weight)	Vita Zahnfabrik; Bad Säckingen, Germany
IPS e.max CAD	Lithium-disilicate glass-ceramic	SiO_2_, Li_2_O, K_2_O, P_2_O_5_, ZrO_2_, ZnO, Al_2_O_3_, MgO and pigments	Ivoclar Vivadent; Schaan, Liechtenstein
IPS e.max ZirCAD	Zirconia ceramic	3 mol.% yttria partially stabilized tetragonal zirconia, ZrO_2_, Al_2_O_3_, Y_2_O_3_	

Aluminum oxide (Al_2_O_3_); 6-ethoxylated-bisphenol-A-dimethacrylate (bis-EMA-6); 2,2-bis[4-methacryloxy polyethoxy)phenyl]propane (bis-MPEPP); bisphenol A diglycidyl ether dimethacrylate (bis-GMA); lithium oxide (LiO_2_); magnesium oxide (MgO); phosphorous pentoxide (P_2_O_5_); polyethylene glycol methacrylate (PEG-DMA); potassium oxide (K_2_O); triethylene glycol dimethacrylate (TEG-DMA); silicon dioxide (SiO_2_); (urethane dimethacrylate (UDMA); zinc oxide (ZnO); zirconium oxide (ZrO_2_); yttrium oxide (Y_2_O_3_).

### Bond Strength Studies

Randomly selected non-carious, non-restored, anonymized human molars, extracted solely for clinical reasons, were collected and kept in distilled water at 4°C for no longer than 6 months. The protocol for this study was reviewed and approved by the Local IRB (protocol # 201709725). Microtensile bond strength (µTBS) and shear bond strength (SBS) tests were performed to evaluate the bonding performance of the different adhesives and resin cements to dental substrates and restorative materials. Sample size for the SBS and µTBS tests was determined based on previous studies.^18,41^

#### Shear bond strength (SBS)

SBS was used to investigate the adhesion of resin cements to different restorative materials as well as the effect of selective enamel etching on the adhesion of composite resin to this substrate. CAD/CAM blocks were used for bonding to restorative materials. For bonding to enamel and dentin, molars were cut in half in a mesio-distal direction in a precision sectioning machine (Isomet 1000, Buehler: Lake Bluff, IL, USA) and embedded in acrylic resin. Initially, all surfaces were flattened with a diamond bur under copious air-water spray using an electric handpiece at 200,000 rpm in a custom-made cutting device (CNC Specimen Former: University of Iowa, Iowa City, IA, USA). Afterwards, LD, 3Y-Zir and 5Y-Zir were sintered according to the respective manufacturer’s recommendations to simulate the sequence and steps of manufacturing a dental restoration. SBS samples were prepared using standardized bonding clamps and mold inserts (Ultradent; South Jordan, UT, USA) with an orifice into which the adhesives were applied on the CAD/CAM blocks, enamel, or dentin surfaces according to manufacturers’ instructions (Table 1) (n = 10). Prior to adhesive application, the surface of 3Y-Zir and 5Y-Zir as well as LU samples were sandblasted with aluminum oxide (50 µm) at 30 psi, cleaned with alcohol, and dried. The LD and VE samples were treated with 5% HF for 20 s and 60 s, respectively, and rinsed. Adhesives were used in self-etching mode for bonding to CAD/CAM blocks and dentin, and both self-etching and selective etching modes were used for enamel. In the selective etching mode, enamel was acid etched with 35% phosphoric acid (Ultra-etch, Ultradent) for 30 s, followed by rinsing with distilled water and drying for 30 s prior to adhesive application. Following adhesive application, specimens were placed on the bonding clamp and the resin cement was inserted. SBU, Estecem II, and RelyX Ultimate were light cured using a Paradigm DeepCure (3M) light-curing unit (operating at 1200 mW/cm^2^ to deliver 12J). In accordance with the manufacturer’s recommendation, SBU was not light cured when used in combination with the resin cement RelyX Ultimate. The bonded specimens were stored in 100% humidity at 37°C for 24 h before testing. The SBS test was performed using the UltraTester testing machine (Ultradent) and results were expressed in MPa.

#### Microtensile bond strength (µTBS)

Molars were mounted in dental stone by their roots, and the crowns were flattened with a carbide bur in an electric handpiece as described for SBS to expose mid-coronal dentin. Adhesives were applied as per the respective manufacturer’s instructions (Table 1) in the self-etching mode, followed by incremental built-up of composite resin, which was light cured as described. After storage in distilled water at 37°C for 24 h, bonded teeth were sectioned using a water-cooled diamond saw mounted in a precision sectioning machine (Isomet 1000, Buehler) to obtain four 2 mm x 2 mm resin-dentin stickes per tooth. Dumbbell-shaped specimens were prepared as previously described^4,41^ and randomly assigned to the different storage conditions: immediate (no aging), long-term storage consisting of 1-year incubation in a medium containing 5 mM HEPES, 2.5 mM CaCl_2_, 0.3 mM NaN_3_, 0.05 mM ZnCl_2_, pH 7.4, at 37°C, replaced every 2 weeks, or thermal cycling (TC) for 100,000 cycles between 5°C and 55°C with a dwell time of 10 s, with the specimens immersed in the same media used for long-term storage (Biometra, Analytik Jena; Jena, Germany) (n = 30). All specimens were tested in tension until failure at a crosshead speed of 1 mm/min in a passive gripping device (Dircks Device; University of Iowa, Iowa City, IA, USA) with a Zwick material-testing machine (Z2.5/TN1S, Zwick/Roell; Ulm, Germany). When two specimens from the same tooth were assigned to the same immediate test or aging, the µTBS values were averaged. After microtensile testing, the failure mode was determined for each debonded specimen using an optical microscope (Stemi 2000, Zeiss; Oberkochen, Germany) and classified as cohesive in composite/dentin, adhesive, or mixed.

#### Statistical Analyses

For the SBS and µTBS data, descriptive statistics were conducted, with means and standard deviations reported for all groups. The comparison of SBS of different restorative materials and dental tissues (enamel and dentin) to resin cements/adhesives was conducted using two-way ANOVA along with one-way ANOVA with a post-hoc Tukey-Kramer test or a two-sample t-test. Regarding bonding to enamel using selective or self-etching mode, two-way ANOVA was performed to determine whether an interaction between the type of adhesive and the method of application existed. If a significant interaction was noted, subsequent analyses for simple effects were conducted using a two-sample t-test to determine whether there was a difference in the bond strength between the two adhesive materials under each aging condition or between the aging conditions within each adhesive material. In addition, when considering tooth dependency, a simple random effect in a mixed-model ANOVA (to allow correlation between specimens from the same tooth) was performed to evaluate the effect of aging conditions on the µTBS. Associations of failure modes with adhesive materials and aging conditions were assessed using Fisher’s exact test. The Shapiro-Wilk test was applied to verify the assumption of normality, as parametric statistical procedures were carried out. For all tests, a significance level of 0.05 was set, and statistical analyses were conducted using the statistical package SAS System, version 9.4 (SAS Institute; Cary, NC, USA).

## Results

**Fig 1:**
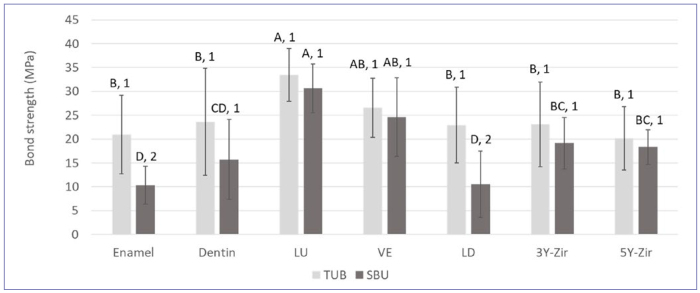
Means and standard deviations of shear bond strength (MPa) of different restorative materials to resin cements and adhesives. TUB: Tokuyama Universal Bond, SBU: Scotchbond Universal, LU: Lava Ultimate, VE: Vita Enamic, LD: IPS e.max CAD lithium disilicate, 3Y-Zir: IPS e.max ZirCAD, 5Y-Zir: Lava Esthetic. Same letters represent no significantly significant difference among substrates (post-hoc Tukey-Kramer test, p > 0.05). Different numbers represent statistically significant difference between resin cements/adhesives within each substrate (two-sample t-test, p < 0.05).

In the comparison of SBS between tooth substrates and restorative materials to the adhesives/resin cements, two-way ANOVA revealed a statistically significant interaction between the adhesives and the different substrates (p = 0.048). When comparing the different resin cements and adhesives for each substrate, the two-sample t-tests showed significant differences in enamel (p = 0.003) and LD only (p < 0.001) (Fig 1), revealing higher bond strengths for TUB/Estecem II than SBU/RelyX Ultimate. For all other substrates (dentin, LU, VE, 3Y-Zir, and 5Y-Zir), adhesives and resin cements performed similarly. When comparing the different substrates with TUB/Estecem, one-way ANOVA followed by the post-hoc Tukey-Kramer test indicated that the SBS observed in LU was significantly higher than that observed in other experimental groups (p < 0.05), except for VE. Moreover, no statistically significant differences were found between LU and VE or among enamel and dentin (with TUB), VE, LD, 3Y-Zir, and 5Y-Zir (p > 0.05 in each instance). Concerning the different substrates for SBU/RelyX, the post-hoc Tukey-Kramer test indicated that the mean SBS observed in LD or enamel was significantly lower than that observed in other experimental groups. Moreover, no statistically significant differences were found between LU and VE or among VE, 5Y-Zir, and 3Y-Zr or among dentin, 5Y-Zr, and 3Y-Zr or between LD and enamel.

**Fig 2:**
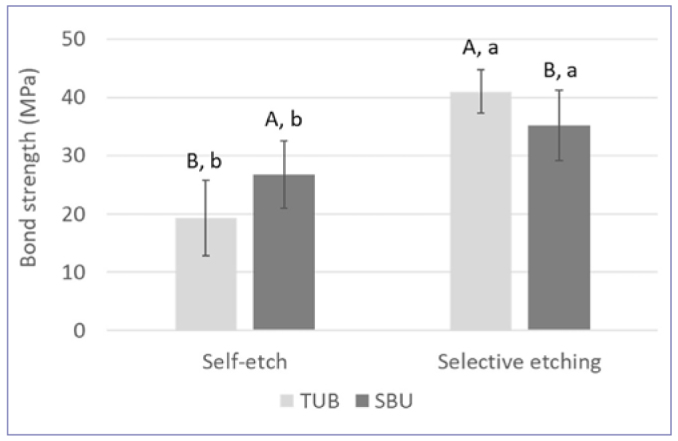
Comparison of resin-enamel shear bond strength (MPa) between adhesives (TUB: Tokuyama Universal Bond, SBU: Scotchbond Universal) and bonding strategies (self-etch and selective etching of enamel). Within each adhesive, different lower-case letters represent statistically significant differences among bonding strategies (p < 0.05). Within each bonding strategy, different upper-case letters represent statistically significant differences among adhesives (p < 0.05).

Concerning the SBS to enamel using selective and self-etching modes, the data demonstrated a significant interaction between adhesive and application mode (p < 0.001), revealing a significant effect for the different adhesives and application modes (Fig 2). Based on the two-sample t-test, when comparing the different modes of application within each adhesive, selective etching resulted in statistically significantly higher enamel bond strength than the self-etching mode (p < 0.001). The comparison between the two adhesives revealed different results depending on the adhesive strategy. More specifically, TUB presented statistically significant higher bond strength than SBU in selective etching mode (p = 0.048), but statistically significantly lower bond strength than SBU when used in the self-etching mode (p = 0.006).

**Fig 3:**
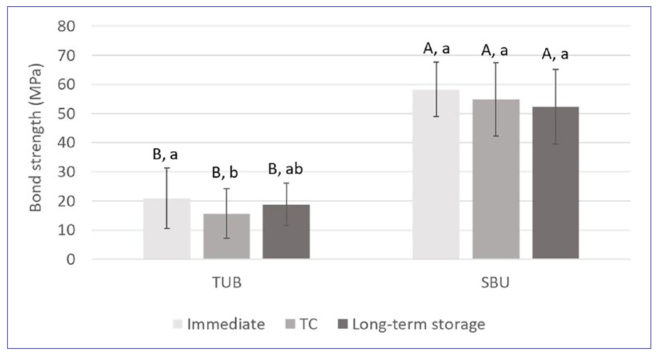
Comparison of resin-dentin microtensile bond strength (MPa) between adhesive systems (TUB: Tokuyama Universal Bond, SBU: Scotchbond Universal) tested immediately, after thermal cycling (100 TC) and after long-term storage (1-year incubation). Within each aging condition, different upper-case letters represent statistically significantly differences between adhesives (p < 0.05). For each adhesive material, different lower-case letters represent statistically significantly differences among aging conditions (p < 0.05).

Regarding the µTBS for the two adhesives, the two-sample t-test showed significant differences between SBU and TUB upon immediate testing, after TC, and after long-term storage (p < 0.001). In all three conditions, SBU exhibited statistically significantly higher bond strength than TUB (Fig 3). The evaluation of the difference among the three aging conditions within each adhesive material (using the mixed-model ANOVA) showed significant differences only for TUB (p = 0.002 for TUB, p = 0.118 for SBU), with immediate bond strengths significantly higher than those after TC (p = 0.016). However, no significant difference was noted in the results between initial and long-term storage or between TC and long-term storage (p > 0.05 in each instance) (Fig 3).

The failure mode data showed no significant association between failure and type of aging condition for both TUB (p = 0.748) and SBU (p = 0.058). In teeth restored with TUB, 96.1% failed adhesively, 2.6% failed cohesively in dentin or resin failure mode, and 1.3% showed a mixed failure mode. When using SBU, 18.5% of the failures were adhesive, 67.9% were cohesive in dentin or resin, and 13.6% were mixed (Table 3). When evaluating failure in each aging condition, there was a significant association at initial testing, after TC and after 1 year of storage (p < 0.001) (Table 4). Regardless of the aging condition, teeth restored using TUB were more likely to have an adhesive (interfacial) failure than those restored with SBU. On the other hand, teeth restored with SBU were more likely to show cohesive failure in dentin or resin than those bonded using TUB for all conditions tested.

**Table 3:** Associations between failure modes and aging conditions for each adhesive material (number of failures n, percentage of failure [%])

Failure modes	Aging conditions			p-value*
	Initial	TC	After 1 year	
For TUB				
Adhesive at interface (n = 73)	27 (96.4%)	24 (96.0%)	22 (95.6%)	0.748
Cohesive in dentin or resin (n = 2)	1 (3.6%)	0 (0.0%)	1 (4.4%)	
Mixed (n = 1)	0 (0.0%)	1 (4.0%)	0 (0.0%)	
For SBU				
Adhesive at interface (n = 15)	8 (26.7%)	5 (20.8%)	2 (7.4%)	0.058
Cohesive in dentin or resin (n = 55)	15 (50.0%)	18 (75.0%)	22 (81.5%)	
Mixed (n = 11)	7 (23.3%)	1 (4.2%)	3 (11.1%)	

*Not statistically significant (p > 0.05) using Fisher’s exact test.

**Table 4:** Association of failure modes and types of adhesive material under each aging condition (number of failures n, percentage of failure [%])

Failure modes	Universal adhesives		p-value*
	TUB	SBU	
Initial (n = 58)			
Adhesive at interface (n = 35)	27 (96.4%)	8 (26.7%)	<0.001
Cohesive in dentin or resin (n = 16)	1 (3.6%)	15 (50.0%)	
Mixed (n = 7)	0 (0.0%)	7 (23.3%)	
TC (n = 49)			
Adhesive at interface (n = 29)	24 (96.0%)	5 (20.8%)	<0.001
Cohesive in dentin or resin (n = 18)	0 (0.0%)	18 (75.0%)	
Mixed (n = 2)	1 (4.0%)	1 (4.2%)	
After 1-year storage (n = 50)			
Adhesive at interface (n = 24)	22 (95.6%)	2 (7.4%)	<0.001
Cohesive in dentin or resin (n = 23)	1 (4.4%)	22 (81.5%)	
Mixed (n = 3)	0 (0.0%)	3 (11.1%)	

*Statistically significant (p < 0.05) using Fisher’s exact test.

## Discussion

According to the present results comparing the adhesion performance of TUB to SBU, TUB was superior to SBU when using resin cements to bond to enamel and LD, while similar SBS results were obtained for the other substrates and CAD/CAM materials (Fig 1). In addition, when comparing the different restorative materials and dental substrates within each adhesive, statistically significant differences were observed, with higher bond strengths for LU and VE (Fig 1). Therefore, the first and second null hypotheses were rejected. When bonding to enamel, selective etching resulted in higher bond strength for both adhesives, and their bonding performance varied according to the bonding strategies (Fig 2); thus, the third null hypothesis is rejected. Lastly, the fourth null hypothesis is also rejected, as SBU adhesion to dentin was superior to TUB, regardless of the aging condition tested (Fig 3).

When interpreting the SBS results comparing SBU/RelyX Ultimate and TUB/Estecem II, the following must be taken into consideration: the effect of the resin cement in the adhesion, and the fact that SBU was not light cured during the bonding procedure, in combination with the resin cement as recommended by the manufacturer. Some resin cements, e.g., RelyX Ultimate, contain an integrated activator for the cement-adhesive polymerization reaction that will result in a “touch cure” or “contact cure” (RelyX Ultimate Technical Data Sheet, 3M Oral Care), in which the chemical reaction starts when the adhesive comes into contact with the resin cement.^5^ If the adhesive is light cured, it could co-polymerize with the cement, which compromises adhesion. On the other hand, when using SBU and RelyX Ultimate for bonding to dentin and comparing the effect of light curing the adhesive and/or resin cement, photopolymerization of both the adhesive and the cement significantly improved the bond strength to dentin in a previous study.^3^ This finding might explain the lower results for SBU than TUB for all restorative materials, enamel, and dentin; however, no statistically significant differences were observed. In fact, for most of the restorative materials (LU, VE, 3Y-Zir, and 5Y-Zir) and dentin, similar results were obtained for SBU and TUB, showing adequate bonding performance of both adhesives to most of the substrates and materials tested here.

Another aspect that should be discussed for the interpretation of the SBS data is the different components in each universal adhesive material. Regarding the silane incorporated in these adhesives, although the manufacturer claims that silane in SBU is stable in a solution with ethanol, filler, and a moderately acidic pH, recent studies revealed that the low pH of SBU (pH = 2.7) may promote hydrolysis and dehydration condensation, thus resulting in the chemical instability of silane.^42,44^ Since TUB is a two-bottle system, it is possible that the γ-meth-acryloxypropyltrimethoxysilane (γ-MPTS) it contains was more stable, since it is separated from water, and the acidic monomer and components are mixed immediately prior to its application. Therefore, the instability of the silane in the SBU adhesive possibly affected the adhesion to some of the indirect materials, particularly LD (Fig 1). Our results agree with other studies that reported reduced bond strength for LD ceramics when a silane agent was not used as a separate step.^1,26^ The hydroxyl groups of the LD and glass matrix should form siloxane bonds due to the reaction with hydrolyzed alkoxy groups in the silane; however, the amount of silane in SBU is unknown, which could be a limitation in this reaction.

On the other hand, both universal adhesives had a positive effect on the SBS of LU and VE. It is possible that the methacrylate monomers from the resin cement may copolymerize with unreacted C=C double bonds from the LU and VE.^37^ Both materials, which are respectively considered a nanoceramic and polymer-infiltrated ceramic, contain methacrylate monomers, as described in Table 2. This is not the case for LD, 3Y-Zir, and 5Y-Zr, in which the bonding mechanism relies on the mechanical roughening, material wettability, and the use of silane coupling agents. Coupling agents are necessary to facilitate the bonding of resin cements to high-strength metal-oxide ceramics, such as zirconia.^29^ Studies have shown that 10-MDP monomer is important for bond stability, and this study demonstrated adequate bond strengths to various substrates when using a universal adhesive with γ-MPTS.

Regarding adhesion to enamel, as expected, selective etching significantly improved the bonding performance of both universal adhesives. The same finding is widely reported in the literature,^10,15,23^ and is explained by the lower potential to promote micromechanical interlocking due to a mild pH of the adhesives and the lower chemical reactivity of acidic monomers like 10-MDP to the hydroxyapatite in enamel.^40,45^ As described in the adhesion-decalcification concept, specific functional monomers can form ionic bonds with hydroxyapatite, resulting in monomer-calcium salts formed via a self-assembling mechanism that forms nano-layers of 10-MDP.^43,45^ While enamel contains more mineral and hydroxyapatite than dentin, the nano-layering formed by 10-MDP at the interface is more intense in dentin than enamel, again justifying the need to etch enamel when using adhesives in the self-etch mode to improve its mechanical interaction with this tissue.^45^ Interestingly, when used in selective enamel etching, TUB performed better than SBU. However, when used in the self-etch mode, SBU resulted in significantly higher bond strength to enamel than TUB (Fig 2). This result indicates that, as its bonding mechanism, TUB might rely more on micromechanical interaction with the enamel than SBU does, in which some chemical interaction of the 10-MDP with the enamel (even if not as strong as with dentin) contributed to the bond strength. Yoshida et al^43^ reported that the formation of calcium-salts by the acidic three-dimensional self-reinforcing (3D-SR) monomer takes longer in enamel than in dentin, which seems to be determined by the differences in crystallites in these two tissues. Although these findings are for the previous generation of the 3D-SR monomer, it supports the SBS results for TUB in comparison to SBU when adhesives were used in self-etching mode.

In fact, it should be highlighted that key differences between the functional monomers present in TUB and SBU may explain their bonding performance to dentin. TUB contains the third generation of 3D-SR, previous versions of which have been used in many adhesives and resin cements from the same manufacturer. Because the chemical structure of 3D-SR presents multiple phosphate groups and polymerizing groups, this monomer is able to form ionic bonds with calcium in dentin at multiple sites, forming three-dimensional cross-links.^29,43^ In addition, when used in dentin, calcium salts formed with 3D-SR monomer proved to be hydrolytically stable, which contributes to bond durability.^43^ These findings agree with the long-term bond strength for TUB, which was similar to the results obtained from the immediate evaluation, but do not explain the decrease in bond strength after thermocycling (Fig 3). Besides the different acidic monomers, the stability of the nano-layering formed by functional monomers depends on the presence of photoinitiators in the adhesive materials when applied to dentin.^45^ Again, based on the resin-dentin bond strength results after thermocycling, it is unclear for TUB if the different polymerization reaction would interfere with the stability of calcium-salts formed by 3D-SR.

Interestingly, regardless of the immediate or simulated aging conditions tested, SBU showed superior performance in bonding to dentin compated to TUB, which can be explained again by differences in their composition. First, the thickness of the nano-layering formed by 10-MDP in dentin (with a periodicity of around 4 nm) is thicker than that reported for the 3D-SR monomer (1.9 nm layer).^43^ Second, the two adhesives contain different solvents. TUB contains isopropyl alcohol, which seems to require a longer evaporation time due to its vapor pressure being lower than ethanol,^17^ which is present in SBU. This was supported by an in-vitro study showing bubble-like areas at the composite/adhesive resin interface bonded with Tokuyama Bond Force, which contains the previous version of the 3D-SR monomer.^6^ The presence of acetone in TUB likely did not affect the bond strength results, as no differences were reported in the bond strength of various light-cured universal adhesives with different solvents.^35^ Third, several in-vitro studies showed good bond strength results, marginal sealing, and stability of interfaces created using SBU.^8,16,24^ Some of the reasons for the good adhesion performance of this material include the presence of Vitrebond copolymer that can result in additional chemical bonding with hydroxyapatite,^34^ the stable nano-layering formed by 10-MDP (as already discussed),^45^ the high adhesive infiltration favored by water displacement by the ethanol used as solvent,^24^ and the capacity to create a thick acid-base resistant zone in dentin interfaces.^29^

## Conclusion

The universal adhesives tested in this study presented different bonding performances depending on the substrate. While SBU showed better adhesion to dentin and enamel when used in the self-etch mode, TUB promoted strong bond strength to enamel in the selective etching mode. When used in combination with resin cements, TUB and SBU universal adhesives presented similar bonding to a nanoceramic, polymer-infiltrated ceramic network, and zirconia. However, the adhesion performance of TUB was superior to SBU when bonding to lithium disilicate. Therefore, clinicians should use caution when selecting a self-curing universal adhesive for direct or indirect restoration, since the performance of this material is substrate dependent.

## Clinical relevance:

In-vitro bonding performance of self-curing and light-curing universal adhesives varies according to the indirect restorative materials or dental substrates. The results of this study emphasize that developing a truly universal material that can used for bonding to several substrates with optimal performance is very challenging and results are material dependent.
